# The function of miRNA in cardiac hypertrophy

**DOI:** 10.1007/s00018-012-1126-y

**Published:** 2012-08-25

**Authors:** Jian Wang, Xiao Yang

**Affiliations:** 1 State Key Laboratory of Proteomics, Genetic Laboratory of Development and Disease, Institute of Biotechnology, 20 Dongdajie, 100071 Beijing, China; 2 Model Organism Division, E-institutes of Shanghai Universities, Shanghai Jiao Tong University, 200240 Shanghai, China

**Keywords:** miRNAs, Cardiac hypertrophy, Signaling pathway, Therapeutic target

## Abstract

Cardiac hypertrophy is an adaptive enlargement of the myocardium in response to altered stress or injury. The cellular responses of cardiomyocytes and non-cardiomyocytes to various signaling pathways should be tightly and delicately regulated to maintain cardiac homeostasis and prevent pathological cardiac hypertrophy. MicroRNAs (miRNAs) are endogenous, single-stranded, short non-coding RNAs that act as regulators of gene expression by promoting the degradation or inhibiting the translation of target mRNAs. Recent studies have revealed expression signatures of miRNAs associated with pathological cardiac hypertrophy and heart failure in humans and mouse models of heart diseases. Increasing evidence indicates that dysregulation of specific miRNAs could alter the cellular responses of cardiomyocytes and non-cardiomyocytes to specific signaling upon the pathological hemodynamic overload, leading to cardiac hypertrophy and heart failure. This review summarizes the cell-autonomous functions of cardiomyocyte miRNAs regulated by different pathways and the roles of non-cardiomyocyte miRNAs in cardiac hypertrophy. The therapeutic effects of a number of miRNAs in heart diseases are also discussed.

## Introduction

The heart is the first organ to form, and becomes functional during vertebrate embryonic development. Embryonic myocardial precursor cells from different origins give rise to different cell types, including cardiac and smooth muscle cells, valvular pacemaker, and endothelial cells, which coordinately build a functional heart [1]. Once developed, the homeostasis of adult heart is maintained by dynamic remodeling in response to altered stress or injury. Upon various mechanical, hemodynamic, hormonal, and pathologic stimuli, the cardiomyocytes initiate a hypertrophic response triggered by a complex cascade of signaling pathways to adapt to stress and improve the function of heart [2]. Compared to the physiologic hypertrophy, which involves proportional increases in the length and width of cardiac myocytes, prolonged concentric or eccentric hypertrophy is usually associated with enhanced synthesis of proteins, assembly of sarcomeres, and both perivascular and interstitial fibrosis, increased expression of embryonic genes including natriuretic peptide and fetal contractile protein genes, and eventually leads to heart failure [3]. Thus, a better understanding of the molecular mechanisms underlying pathological hypertrophy will greatly benefit rational drug development for heart failure treatment.

Recently, increasing evidence has revealed that microRNAs (miRNAs) play important regulatory roles in cardiac development and disease [4, 5]. MiRNAs are endogenous small non-coding RNAs of 18–25 nt in length. They exert biological functions by post-transcriptional regulation of gene expression in a sequence-specific manner. MiRNAs are transcribed mainly by RNA polymerase II as pri-miRNAs that are usually several thousand bases in length. The pri-miRNAs are subsequently processed in the nucleus into a 70–100 nt hairpin RNAs (pre-miRNAs) by the RNase III-type enzyme Drosha, and cleaved by Dicer in the cytoplasm, to form the mature double-stranded miRNAs [6]. One strand of the mature miRNA is incorporated into the miRNA-induced silencing complex (miRISC) to bind target mRNA through its seed sequence. Binding of mature miRNAs to mRNAs usually results in the repression of target gene expression by either degrading the target mRNA or inhibiting the translation [6]. Each miRNA could repress up to hundreds of transcripts, and it is thus hypothesized that miRNAs form large-scale regulatory networks across the transcriptome through miRNA response elements (MREs) [7].

MiRNAs are differentially and temporally regulated during cardiac hypertrophy and heart failure. In vivo gain- and loss-of-function miRNA studies in mouse have demonstrated physiological and pathogenic roles of miRNAs in cardiac hypertrophy. Most importantly, in vivo manipulation of miRNAs by a specific antagomir or mimic provides new opportunities for therapeutic treatment for cardiac hypertrophy and heart failure. This review describes the biological functions and mechanisms of miRNAs in cardiac hypertrophy, and highlights the possibility for miRNAs as therapeutic targets for cardiac hypertrophy.

## Dysregulation of miRNAs in cardiac hypertrophy

The dysregulation of miRNAs has been demonstrated in cardiac hypertrophy by a series of high-throughput miRNA microarray analyses [8–11]. The earliest array study was performed based on two mouse models of pathological hypertrophy: the transverse aortic constriction (TAC) mouse model, an in vivo model of hypertrophy induced by left ventricular pressure-overload, and the calcineurin transgenic mouse model, a calcium-dependent model of maladaptive response. The results showed that a specific group of miRNAs were similarly dysregulated in both models. The altered pattern of miRNA expression in the hypertrophic mouse heart largely mimics that of the idiopathic end-stage failing human heart, indicating that specific miRNAs represent a molecular signature of cardiac hypertrophy and might have critical roles during the pathological process [8]. Further miRNA profiling studies revealed that the expression of the dysregulated miRNAs progressively changes during development of pressure-overload cardiac hypertrophy [9].

MiRNA array analyses also demonstrate that the alterations of a set of fetal miRNAs substantially contribute to reactivation of fetal gene programs in the failing human heart [10]. The altered expression of miRNAs in failing hearts closely resembles the miRNA expression signature observed in fetal cardiac tissue. A significant inverse correlation exists between miRNAs and their regulated mRNA expression in heart failures. Overexpression of specific fetal miRNAs in cultured cardiomyocytes triggers fetal gene expression and induces cellular hypertrophy, suggesting that altered miRNAs might have important roles in initiating cardiac hypertrophy.

MiRNA expression profiling studies have revealed novel miRNA-based pathways underlying the cardiac diseases. One piece of evidence from bioinformatics shows that the eight dysregulated miRNAs in human heart failure might affect 1,716 predicted targets, which associate with diverse signaling networks [11]. The results suggest that cardiac miRNAs are integrated into complex regulatory networks, and dysregulation of miRNAs can affect multiple cellular processes in ways that are conducive to the establishment of cardiac diseases.

## Cell-autonomous function of miRNAs in cardiac hypertrophy

**Fig. 1:**
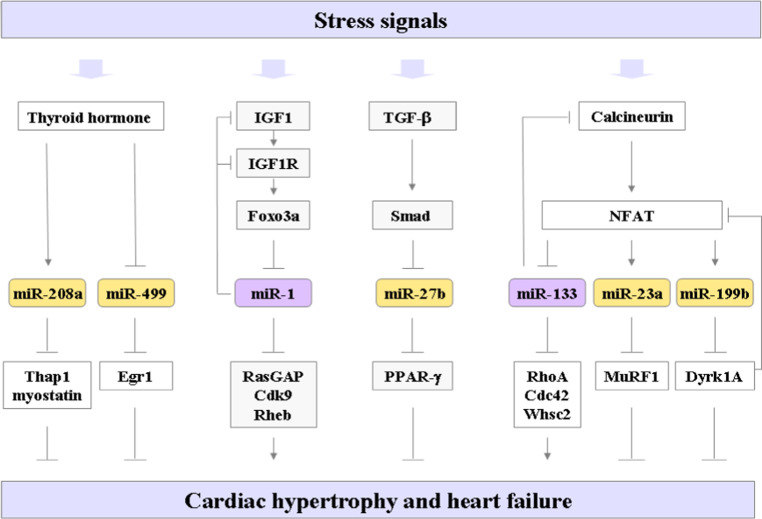
Cell-autonomous function of cardiomyocyte miRNAs in cardiac hypertrophy. Multiple signaling pathways, including the thyroid hormone, IGF-1, TGF-β, and calcineurin cascades control the hypertrophic response of cardiomyocytes in the heart in response to various stresses. The miRNAs regulated by these signaling pathways are depicted

Various injuries and stresses stimulate a pathological gene response and hypertrophic growth in adult cardiomyocytes, often leading to heart failure and sudden death. Altered cellular responses of cardiomyocytes to specific signaling pathways might eventually hamper the cardiac performance in the failing heart. The molecular mechanisms underlying cardiac hypertrophy and heart failure have been extensively studied, and many pathways involved in these processes have been uncovered, such as thyroid hormone, IGF1/Akt, calcineurin/NFAT and TGF-β/Smad-signaling pathways. Recent studies have shown that miRNAs regulated by these signaling pathways are involved in the process of cardiac hypertrophy (Fig. 1).

### MiRNAs regulated by thyroid hormone

Thyroid hormones have complex and important effects on the cardiovascular system [12]. Increased thyroid hormones can induce physiological hypertrophy characterized by increased gene encoding of the calcium pump of the sarcoplasmic reticulum (SERCa2), increased myosin heavy chain α (α-MHC, a fast myosin) levels, and decreased β-MHC levels, while a downregulation of the thyroid hormone signaling system is associated with concentric cardiac hypertrophy and heart failure [13]. Recently, a family of miRNAs (miR-208a, miR-208b, and miR-499) encoded by MHC genes, has been shown to form an intricate regulatory circuit together with their host genes to regulate cardiac hypertrophy. These miRNAs are co-expressed and are regulated together with their host MHC genes by the same transcriptional events and signaling pathways. Treatment of thyroid hormones significantly upregulates α-MHC/miR-208a expression and reduces β-MHC/miR-208b expression [14]. miR-208a is a cardiac-specific miRNA encoded by intron 27 of the α-MHC gene and regulates two slow myosins and their intronic miRNAs, β-MHC/miR-208b and Myh7b/miR-499. miR-208b is encoded within intron 31 of β-MHC gene, while miR-499 is encoded by intron 19 of the mouse Myh7b gene, which shares high homology with the β-MHC gene [15]. miR-208b and miR-499 play redundant roles in the specification of muscle fiber identity by activating slow myofiber and repressing fast myofiber gene programs. Targeted disruption of miR-208a in mice diminishes the re-expression of the fetal β*-*MHC gene in response to stress and hypothyroidism, and protects the heart from pathological hypertrophy [16]. Consistently, transgenic overexpression of miR-208a in the heart induces hypertrophic growth in mice by suppressing thyroid hormone-associated protein 1 and myostatin, which are negative regulators of muscle growth and hypertrophy [14]. Elevated levels of miR-499 in the heart alter the cardiac response to stress in part by modulating the immediate early response genes, and lead to cardiomyocyte hypertrophy and cardiomyopathy in a dose-dependent manner, especially in the setting of pressure overload [17]. These studies have demonstrated that specific miRNA upregulation can lead to cardiac hypertrophy caused by dysregulation of thyroid hormone signaling.

### MiRNAs regulated by IGF-1 signaling

The IGF-1/phosphoinositide 3-kinase (PI3K)-Akt signaling plays an important function during physiological cardiac remodeling [18]. To initiate the intracellular response to IGF-1 signaling, activated intrinsic receptor tyrosine kinases phosphorylate a number of intracellular substrates such as the insulin receptor substrate-1 and Shc, which recruit and activate downstream signaling pathways including PI3K/Akt cascades. Dysregulation of IGF-1 signaling has also been involved in pathological hypertrophy [19]. Recent studies have revealed that miRNAs play critical roles in mediating the effects of the IGF-1 pathway in physiological and pathological conditions of heart. MiR-1 and miR-133 have been shown to be down-regulated in exercised trained rats and cardiac-specific Akt transgenic mice, which are models of physiological cardiac hypertrophy [20, 21]. The miR-1 has been further demonstrated to be down-regulated by IGF-1 stimulation depending on the Foxo3 transcription factor. Reciprocally, miR-1 controls the expression of IGF-1 and IGF-1 receptor protein levels by translation. Importantly, miR-1 expression correlates inversely with overproduced growth hormone and IGF-1, as well as dramatically increased cardiac myocyte size in cardiac biopsies of patients with acromegaly, demonstrating the clinical relevance of a feedback loop between miR-1 expression and the IGF-1 signal transduction pathway in human cardiac hypertrophy [22]. Consistently, liver-specific IGF-1-deficient mice resist abdominal aortic constriction (AAC)-induced cardiac hypertrophic and contractile changes via alleviating down-regulation of miR-1 and miR-133a [23].

### MiRNAs regulated by TGF-β signaling

The transforming growth factor-β (TGF-β) superfamily contains more than 40 ligands, which signal through receptor serine/threonine kinases and intracellular Smad proteins. The role of TGF-β signaling in cardiac hypertrophy has been extremely contradictory due to the complexity of TGF-β activation as well as its diverse effects on different type of cells. Although TGF-β1 has been considered as detrimental, inducing cardiac hypertrophy and failure in the adult heart [24, 25], increasing evidence shows that endogenous TGF-β/Smad signaling protect cardiomyocyte from hypertrophic growth [26–29]. We have previously shown that targeted deletion of Smad4, the central intracellular mediator of TGF-β superfamily signaling, in cardiomyocyte, unexpectedly leads to cardiac hypertrophy [26]. For the first time, the result provides in vivo genetic evidence demonstrating that the endogenous cardiomyocyte Smad4-dependent TGF-β pathway protects heart from cardiac hypertrophy and fibrosis. This observation also suggests that a lack of responsiveness to TGF-β could be a new mechanism of myocardial hypertrophy and heart failure. This hypothesis has been supported by other studies showing that other members of the TGF-β superfamily play an antihypertrophic role through the Smads in the heart [27–29]. Transaortic constriction leads to a significant increase in cardiac hypertrophy in the Smad3 knockout mice, indicating a beneficial role of Smad3 by delimiting cardiomyocyte hypertrophic growth [27]. Growth differentiation factor 15 (GDF15) has been demonstrated to have antihypertrophic and cardioprotective effects via activating Smad2/3 [28]. In addition, cardiac-specific overexpression of BMP-10 also impairs postnatal cardiac hypertrophic growth by activating Smad1/5/8 [29]. All these results suggest that endogenous canonical TGF-β/Smad signaling of cardiomyocyte is required for maintaining cardiac homeostasis and preventing cardiac hypertrophy.

Recently, we have revealed that the function of endogenous TGF-β/Smad signaling in maintaining cardiac homeostasis involves the downregulation of miRNAs inducing cardiac hypertrophy. We show that TGF-β1 inhibits the expression of miR-23a/miR-27a/miR-24-2 and miR-23b/miR-27b/miR-24-1 clusters at the transcriptional level [30, 31]. Overexpression of miR-27b promotes hypertrophic cell growth, whereas knockdown of miR-27b leads to inhibition of the hypertrophic cell growth caused by phenylephrine (PE) treatment in vitro. In addition, transgenic mice with cardiomyocyte-specific overexpression of miR-27b exhibit cardiac hypertrophy and dysfunction by directly targeting the peroxisome proliferator-activated receptor-γ (PPAR-γ). These results provide the first, and critical, genetic evidence showing that TGF-β-regulated miR-27b plays an important role in cardiac hypertrophy [30]. TGF-β/Smads signaling might elicit its function in preventing cardiac hypertrophy by down-regulating other miRNAs. MiR-24 has been demonstrated to play an important role in participating TGF-β-induced inhibition of myogenesis. Ectopic expression of miR-24 enhances myogenic differentiation and partially rescued TGF-β-inhibited myogenesis in the C2C12 myoblast cell line, while knockdown of miR-24 results in the downregulation of myogenic differentiation markers [31]. MiR-24 is up-regulated during cardiac hypertrophy from mice in response to transverse aortic constriction or expression of activated calcineurin, as well as in idiopathic end-stage failing human hearts, and overexpression of miR-24 induces hypertrophic growth of cardiomyocyte in vitro [8]. However, whether the down-regulation of miR-24 plays a function in TGF-β regulated cardiac homeostasis needs to be further demonstrated.

### MiRNAs regulated by calcineurin signaling

Calcineurin signaling is a major intracellular regulator of cardiomyocyte growth and function. Activation of the calcineurin and its downstream targets, transcriptional effector nuclear factor of activated T-cells (NFATs), results in cardiac hypertrophy. In a study screening for miRNAs regulated during cardiac hypertrophy and heart failure, the transgenic mice expressing activated calcineurin A show significantly altered expression of miRNAs, suggesting that miRNA could mediate calcineurin signaling to modulate the cardiac hypertrophy [8].

MiR-23a has been shown to be upregulated in calcineurin A transgenic mice and upon the treatment with the hypertrophic stimuli including isoproterenol and aldosterone [8, 32]. MiR-23a is up-regulated by NFATc3 at the transcriptional level, and able to convey the calcineurin/NFATc3-mediated hypertrophic signal by directly targeting the muscle-specific ring finger protein 1, an anti-hypertrophic protein.

Reciprocal repression between microRNA-133 and calcineurin signaling has been shown to regulate cardiac hypertrophy. Cardiac hypertrophy in vivo and in vitro induced by transverse aortic constriction and phenylephrine (PE) treatment involves increased activity and expression of calcineurin and decreased miR-133 expression. Treatment of neonatal rat cardiomyocytes or mice with cyclosporin A, an inhibitor of calcineurin, prevents the down-regulation of miR-133. Consistently, antisense oligonucleotides against the catalytic subunit of calcineurin A and NFAT-specific decoy oligonucleotides could increase miR-133 expression in cultured primary cardiomyocytes [33]. Gain-of-function approaches also show that miR-133 decreases NFAT mRNA levels as well as the hypertrophic response to PE-mediated stimulation in primary cardiomyocytes, and miR-133 loss-of-function leads to increased NFAT expression and a spontaneous hypertrophic response [33].

Recently, miR-199b, which is upregulated in mouse and human heart failure, has been demonstrated to modulate calcineurin-NFAT signaling-mediated cardiac hypertrophy in a positive feedback loop [34]. Calcineurin induces the expression of miR-199b through a functional NFAT site about ~2.7 kb upstream of the miR-199b gene. MiR-199b directly targets the dual-specificity tyrosine (Y) phosphorylation-regulated kinase 1a (Dyrk1a), which has been previously shown to prevent nuclear occupancy of NFATc transcription factors [35]. Transgenic mice overexpressing miR-199b, or haploinsufficient for Dyrk1a, exhibit more exaggerated cardiac hypertrophic phenotype induced by calcineurin/NFAT signaling or pressure overload. In vivo inhibition of miR-199b by a specific antagomir normalizes Dyrk1a expression, reduces nuclear NFAT activity, inhibits and even reverses the cardiac hypertrophy and fibrosis in mouse models of heart failure [34].

All these results indicate that miRNAs regulated by calcineurin can affect cardiac cellular calcineurin signaling and cardiac homeostasis.

## Non-cardiomyocyte miRNAs in cardiac hypertrophy

In addition to cardiomyocytes, the heart contains many other “non-cardiomyocyte” cell types, such as fibroblasts, endothelial cells, smooth muscle cells, and immune cells, which may have completely distinct miRNA and target gene expression profiles. These non-cardiomyocytes play important roles in both cardiac hypertrophy and heart failure by stimulating complex cardiac remodeling, which involves interstitial fibrosis, angiogenesis, and inflammation. Recent studies have revealed the essential function of non-cardiomyocyte miRNAs in maintaining cardiac homeostasis by regulating cardiac fibrosis and inflammation.

### TGF-β signaling-related miRNAs and cardiac fibrosis

Fibroblasts that represent the majority of non-cardiomyocytes in heart are essential for the cardiac adaptive response to pressure overload [36]. Cardiac fibroblasts produce extracellular matrix (ECM) proteins, as well as paracrine factors that can regulate the function of cardiomyocytes. Reciprocally, the function of fibroblasts could be modulated by paracrine factors secreted by cardiomyocytes. TGF-β1 is the most important cytokine that contributes to fibrotic response. It induces fibroblasts to produce ECM and reduce collagenase production, resulting in an overall inhibition of ECM degradation and leading to excessive matrix accumulation. Recent studies reveal that fibroblastic miRNAs regulated by TGF-β signaling contribute to cardiac hypertrophy.

MiR-21 is among the most strongly upregulated miRNAs in response to a variety of forms of cardiac stresses [37, 38], and induced by TGF-β signaling through a post-transcriptional step [39, 40]. MiR-21 is specifically upregulated in cardiac fibroblasts in the failing heart. Upregulation of miR-21 in cardiac fibroblasts targets the sprouty homologue 1 (Spry1), thereby indirectly enhancing the activity of the mitogen-activated protein kinase (MAPK)/extracellular signal-regulated kinase (ERK) signaling pathway. This positively regulates cardiac fibroblast survival, leading to fibrosis, hypertrophy, and cardiac dysfunction [41]. The fibrotic function of miR-21 has been further confirmed by later studies in models of pulmonary and renal fibrosis [42, 43]. However, there is obviously contradictory data about the function of miR-21 in stress-dependent cardiac remodeling. miR-21-deficient null mice have been reported to display cardiac hypertrophy and fibrosis comparable to wild-type littermates in response to various cardiac stresses, suggesting that miR-21 is not essential for pathological cardiac remodeling [44]. MiR-21 has also been shown to induce the expression of matrix metalloproteinase-2 by targeting the phosphatase and tensin homolog (PTEN) in the fibroblasts [45].

The miR-29 family is composed of three members, miR-29a, b and c, which are preferentially expressed in cardiac fibroblasts as compared with cardiomyocytes and downregulated in the mouse heart in response to TAC, chronic calcineurin signaling, and in the viable myocardium after myocardial infarction (MI) [46]. Interestingly, miR-29 is downregulated after TGF-β stimulation in cultured cardiac fibroblast, and upregulated in Smad3 knock-out heart, suggesting that down-regulation of miR-29 could contribute to TGF-β-induced fibrosis. Studies also show that the miR-29 family targets mRNAs encoding a multitude of ECM-related proteins involved in fibrosis, including multiple collagens, fibrillins, and elastin. Downregulation of miR-29 with anti-miRs in vitro and in vivo induces the expression of collagens, whereas overexpression of miR-29 in fibroblasts reduces collagen expression. All these data indicate that miR-29 acts as a regulator of cardiac fibrosis, and might serve as a potential therapeutic target for cardiac fibrosis [46].

MiR-133 and miR-30 have been shown to inhibit cardiac fibrosis by targeting and connective tissue growth factor (CTGF). CTGF is considered a key molecule in inducing ECM synthesis, and positively regulated by TGF-β [47]. MiR-30 is highly expressed in cardiac fibroblasts, and may decrease the production of collagens by directly targeting CTGF. Knockdown of miR-30c in cultured fibroblasts increases CTGF expression, whereas overexpression of miR-30 decreases CTGF. The change of CTGF expression induced by the miR-30 is accompanied by parallel changes in collagen expression in fibroblasts. Importantly, a reduction in miR-30 expression inversely correlates with CTGF in rodent models of cardiac hypertrophy and samples from patients with left ventricular hypertrophy [48]. Although miR-133 is expressed mainly in cardiomyocyte, its function in regulating fibrosis has been demonstrated by in vitro and in vivo experiments [49, 50]. Cardiomyocyte-specific overexpression of miR-133a in mice leads to reduced apoptosis and fibrosis after TAC, while knockout of the two miR-133a genes results in dilated cardiomyopathy accompanied by extensive fibrosis in surviving mutant mice. Mechanistically, miR-133a inhibits cardiac fibrosis by directly targeting CTGF, which regulate the myocardial matrix remodeling through a paracrine mechanism.

### NF-κB regulated miRNA and cardiac inflammation

Accumulated evidence indicates an important role for inflammation in cardiac hypertrophy and failure. Patients with HF have elevated levels of a number of inflammatory cytokines both in the circulation and in the failing heart itself. Inflammatory cytokines such as tumor necrosis factor-α (TNF-α), interleukin-1β (IL-1β), NF-κB and monocyte chemoattractant peptide-1 (MCP-1) may contribute to the development and progression of HF by promoting myocardial hypertrophy, activating matrix metalloproteinases, provoking contractile dysfunction, and inducing apoptosis [51].

NF-κB, the main downstream effector molecule of TLR (Toll-like receptors) signaling, is a nuclear transcription factor that increases the expression of proinflammatory cytokines like TNF-α, IL-1, IL-6, and IFN-γ. Persistent activation of proinflammatory cytokine expression appears to have detrimental effects on heart. In human heart failure patients, increased NF-κB activation has been detected [52]. Whereas chemical NF-κB inhibition largely decreases myocardial infarct size in rats, indicating a detrimental role of NF-κB in stressed hearts [53]. These results suggest that NF-κB is a crucial component in the immune response that occurs during the development of cardiac hypertrophy and heart failure.

MiR-146 is abundantly expressed in the heart and is upregulated upon activation of NF-κB [54]. Considering that miR-146 is rapidly induced mediated by TLR and NF-κB in immune cells upon lipopolysaccharides (LPS) exposure [54], and miR-146 is increased in the cardiomyocyte-specific Dicer-deficient mice that showed signs of inflammation, this induction probably originates from inflammatory cells that invade the myocardium [4]. The increased miR-146 leads to the downregulation of two downstream mediators of TLR signaling- IRAK and TRAF6, creating an anti-inflammation feedback loop to inhibit NF-κB-induced proinflammatory cytokine production. In addition, miR-146 is found to be upregulated in mouse models of MI and TAC [55], implicating that this miRNAs is involved in the pathogenesis of HF by influencing the inflammatory response. Until now, the role for miR-146 in heart disease has been unknown; further studies should be performed to determine its function in the pathogenesis of cardiac hypertrophy and heart failure.

## miRNAs as potential therapeutic targets in cardiac hypertrophy

The identification of miRNAs as important regulators in cardiac hypertrophy raises their therapeutic implications in cardiac diseases. To date, several tools are available to selectively target miRNA pathways. Chemically engineered oligonucleotides, termed “mimic” and “antagomirs”, and adenovirus that expresses specific sense or antisense miRNAs, have been developed and evaluated for their therapeutic effect on cardiac diseases. Until now, a number of miRNAs have been proven to be effective therapeutic targets for cardiac diseases.

### Cardiomyocyte miRNAs act as therapeutic targets

Dysregulation of cardiomyocyte miRNAs disturbs cardiac homeostasis by disrupting the cellular responses of cardiomyocytes to various signaling pathways. Recent studies have demonstrated that re-expression of downregulated anti-hypertrophic miRNAs or knockdown of upregulated pro-hypertrophic miRNAs is able to modulate cardiac remodeling, and serve as a promising therapeutic approach.

## In vivo overexpression of anti-hypertrophic miRNAs attenuates cardiac hypertrophy

MiR-133 is decreased in mouse and human models of cardiac hypertrophy, and has been shown to inhibit cardiac hypertrophy by targeting RhoA, Cdc42, and Nelf-A/WHSC2. To examine whether miR-133 overexpression has a beneficial effect on cardiac hypertrophy, adenovirus expressing miR-133 (Ad133) infection is performed in Akt transgenic mice that develop significant cardiac hypertrophy. Overexpression of miR-133 results in a significant reduction in the size of left ventricular cardiac myocytes and a significant decrease in the expression of fetal genes, indicating that miR-133 acts as a therapeutic target for cardiac hypertrophy [21]. However, another study shows that miR-133a overexpression in postnatal cardiomyocytes does not alter reactive hypertrophy in pressure-overloaded or isoproterenol-treated hearts, although it protects against myocardial fibrosis [50]. The discrepancy indicates that miR-133a might have a defined range of expression that is required for preventing cardiac hypertrophy, and suggest that for any miRNA-directed therapeutic approach may have to face a narrow therapeutic window.

MiR-98/Let-7 has been demonstrated to mediate the antihypertrophic effect of thioredoxin (Trx1) [56]. Studies show that Trx 1 negatively regulates Ang II-induced cardiac hypertrophy through upregulation of miR-98/let-7. To evaluate the function of miR-98 in the heart in vivo, Ad-miR-sc, Ad-miR-98, or Ad-anti–miR-98 has been injected into mouse hearts, following which mice have been treated with or without continuous infusion of Ang II. Although neither Ad-miR-98 nor Ad-anti–miR-98 significantly affects heart morphology at baseline, Ang II–induced increases in LVW/BW, LV myocyte cross-sectional area, cardiac fibrosis, and apoptosis are significantly attenuated by Ad-miR-98 and significantly enhanced by Ad-anti–miR-98. These results suggest that miR-98 inhibits Ang II–induced cardiac hypertrophy and might be a therapeutic target for cardiac diseases [56].

## In vivo inhibition of pro-hypertrophic miRNAs attenuates cardiac hypertrophy

MiR-23a has been shown to be able to convey the hypertrophic signal of ISO stimulation. To test whether knockdown of miR-23a in vivo can be benefit for cardiac hypertrophy, antagomir-23a has been delivered into heart of mice perfusion with ISO. Antagomir-23a inhibits ISO-induced hypertrophy revealed by heart weight/body weight ratio, hypertrophic phenotype, cross-sectional areas, the expression levels of hypertrophic markers, as well as cardiac size and function. These data suggest the therapeutic prospect of miR-23a in cardiac hypertrophy [32].

Cardiac-specific knockout of miR-208a prevents cardiomyocyte hypertrophy or fibrosis upon various cardiac stresses, suggesting that inhibition of miR-208a might serve as a therapeutic strategy [16]. The therapeutic potential of miR-208a has been demonstrated by a recent study, in which a locked nucleic acid (LNA)-modified antisense oligonucleotide against miR-208a is delivered [57]. The inhibition of miR-208a during hypertension-induced heart failure in Dahl-hypertensive rats significantly diminishes stress-induced remodeling, prevents pathological myosin switching, and improves cardiac function, overall health, and survival [57].

Overexpression of miR-199b sensitizes the myocardium to pathological cardiac hypertrophy [34]. To test whether antagomir-199b can prevent or cure cardiac disease, wild-type and calcineurin transgenic mice are subjected to TAC and then treated intraperitoneally with antagomir-199b on three consecutive days at the age of 14 days after birth. TAC-induced interstitial fibrosis, cardiac hypertrophy, and left ventricular dilatation are significantly prevented or attenuated in mice administrated antagomiR-199b, and fractional shortening and systolic and diastolic contractile defects are also normalized, implicating that miR-199b could serve as a therapeutic target for heart failure [34].

Our recent work demonstrate the therapeutic potential of miR-27b for cardiac disease. We have injected antagomir-27b into mice subjected to pressure overload of the left ventricle by TAC. Expression levels of miR-27b are increased in mice after the TAC surgery, and are effectively downregulated by antagomir-27b treatments. Antagomir-27b treatments significantly reverse changes in its target gene PPAR-γ expression caused by pressure overload in the myocardium, and attenuate TAC-induced cardiac hypertrophy, interstitial fibrosis, and cardiac dysfunction, suggesting that miR-27b is a novel therapeutic target for pathological cardiac hypertrophy [30].

### Non-cardiomyocyte miRNAs as therapeutic targets

miR-21 has been shown to regulate fibroblast survival and growth factor secretion to control the extent of interstitial fibrosis and cardiac hypertrophy, raising the possibility that inhibition of miR-21 expression in a cardiac disease model should prevent and cure fibrosis. For example, to test the preventive and curative potential of silencing of miR-21, antagomir-21 was injected into mice subjected to pressure overload of the left ventricle by TAC [41]. Of interest, inhibition of miR-21 significantly attenuates interstitial fibrosis, enlarged cardiomyocyte size, cardiac dysfunction and normalizes the expression of various genes that are dysregulated after TAC [41], implicating the therapeutic effect of miR-21 in a cardiac disease setting. However, another study shows that inhibition of miR-21 by a LNA-modified antimiR oligonucleotide fails to block the remodeling response of the heart to various stresses, indicating miR-21 is not essential for pathological cardiac remodeling [44]. The discrepancy between these two studies may be due to the different inhibitors and different delivery methods [58], and this needs to be further clarified in the future.

## Conclusions and perspectives

Recent studies provide clear evidence that miRNAs play important roles by fine-tuning gene expression in response to physiological or pathological stress. A number of signaling cascades related with cardiac hypertrophy can modulate hypertrophic response by regulating the expression of cardiomyocyte and non-cardiomyocyte miRNAs. More importantly, miRNAs and their regulated signaling pathways have been demonstrated to be valuable potential therapeutic targets for cardiac diseases. Nevertheless, further investigation is needed to determine how the binding of multiple miRNAs affects the expression of individual targeted mRNAs, and how multiple miRNA targets are interlinked to affect the various pathways and cardiac remodeling. Recently, a new concept- “competing endogenous RNA (ceRNA)” has been proposed to explain how different types of RNAs, including long non-coding RNAs, “talk” to each other using miRNA response elements (MREs) as letters of a new language [7]. Hence, further investigation on the function of miRNAs and their regulated networks may help to decipher the complex mechanism underlying cardiac hypertrophy and heart failure, and as a result, it may provide new therapeutic opportunities for this disease.
